# Green fluorescent protein fused to peptide agonists of two dissimilar G protein-coupled receptors: novel ligands of the bradykinin B_2_ (rhodopsin family) receptor and parathyroid hormone PTH_1_ (secretin family) receptor

**DOI:** 10.1002/prp2.4

**Published:** 2013-10-04

**Authors:** Xavier Charest-Morin, Jean-Philippe Fortin, Marie-Thérèse Bawolak, Robert Lodge, François Marceau

**Affiliations:** 1 Centre de recherche en rhumatologie et immunologie, CHU de Québec, Université LavalQuébec, Canada, G1V 4G2; 2 Pfizer's Cardiovascular and Metabolic Diseases Research UnitCambridge, Massachusetts, 02139; 3 Laboratory of Human Retrovirology, Institut de recherches cliniques de MontréalMontreal, Québec, Canada, H2W 1R7

**Keywords:** Biochemical pharmacology, bone pharmacology, ligands, vascular pharmacology

## Abstract

We hypothesized that peptide hormone sequences that stimulate and internalize G protein-coupled receptors (GPCRs) could be prolonged with a functional protein cargo. To verify this, we have selected two widely different pairs of peptide hormones and GPCRs that nevertheless share agonist-induced arrestin-mediated internalization. For the parathyroid hormone (PTH) PTH_1_ receptor (PTH_1_R) and the bradykinin (BK) B_2_ receptor (B_2_R), we have designed fusion proteins of the agonists PTH_1-34_ and maximakinin (MK, a BK homologue) with the enhanced green fluorescent protein (EGFP), thus producing candidate high molecular weight ligands. According to docking models of each hormone to its receptor, EGFP was fused either at the N-terminus (MK) or C-terminus (PTH_1-34_) of the ligand; the last construction is also secretable due to inclusion of the preproinsulin signal peptide and has been produced as a conditioned medium. EGFP-MK has been produced as a lysate of transfected cells. Using an enzyme-linked immunosorbent assay (ELISA) for GFP, average concentrations of 1.5 and 1670 nmol/L, respectively, of ligand were found in these preparations. The functional properties and potential of these analogs for imaging receptor-expressing cells were examined. Microscopic and cytofluorometric evidence of specific binding and internalization of both fusion proteins was obtained using recipient HEK 293a cells that expressed the cognate recombinant receptor. Endosomal colocalization studies were conducted (Rab5, Rab7, β-arrestin_1_). Evidence of agonist signaling was obtained (expression of c-Fos, cyclic AMP responsive element (CRE) reporter gene for PTH_1-34_-EGFP). The constructs PTH_1-34_-EGFP and EGFP-MK represent *bona fide* agonists that support the feasibility of transporting protein cargoes inside cells using GPCRs.

## Introduction

G protein-coupled receptors (GPCRs) correspond to numerous (>800) and diverse genes in mammalian genomes (Fredricksson and Schiöth 2005; Nordström et al. 2011). The five larger phylogenetic groups of GPCRs are only distantly related, but they share their 7-transmembrane domain tertiary structure, general signaling pattern as guanine nucleotide exchange factors for heterotrimeric G proteins and, for many, desensitization mechanisms such as receptor phosphorylation, association with β-arrestins and endocytosis. We aim at evaluating the feasibility of the transport of functional cargoes by agonists of receptors for peptide hormones that activate arrestin-dependent endocytosis (Bawolak et al. 2011, 2012; Gera et al. 2012, 2013).

The parathyroid hormone (PTH) is an 84 aminoacid peptide secreted by chief cells of the parathyroid glands. PTH_1-84_, mainly via its G protein-coupled PTH_1_ receptor (PTH_1_R) expressed in kidneys and the skeleton, is an important regulator of calcium metabolism and bone formation (Datta and Abou-Samra 2009) (receptor nomenclature as in Alexander et al. 2011). Structure–function studies support that the N-terminal fragment 1–34 of the PTH_1-84_ chain is sufficient to bind and fully activate PTH_1_R (Vilardaga et al. 2012). This idea is consistent with the observation that synthetic PTH_1-34_ (also named teriparatide) exerts potent antiosteoporosis actions in humans and the osteoblast constitutes the target cell type for this action mediated by PTH_1_Rs (Kousteni and Bilezikian 2008; Pullenayegum et al. 2011).

**Figure 1:**
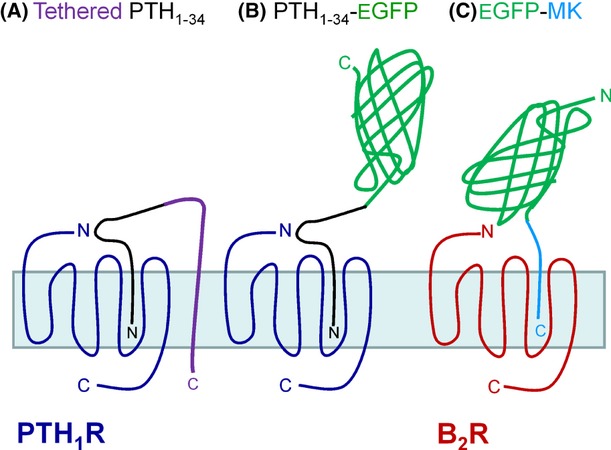
(A, B) Schematic representation of the PTH_1_R and its biotechnological ligands (tethered or GFP conjugated) discussed or exploited in the present text. (C) Schematic representation of the BK B_2_R and its GFP-conjugated ligand based on maximakinin (MK).

As with other peptide agonists of the secretin receptor family (“class B”), studies support that PTH_1_R is activated via a two-domain model (Hoare 2005), whereby the C-terminal helical portion of PTH_1-34_ initially binds the large extracellular domain of the receptor. Secondly, the N-terminal residues of the hormone interact with the receptor juxtamembrane domain (transmembrane helices and connecting extracellular loops), thus triggering the activation of intracellular signaling pathways. PTH_1_R is also subjected to agonist-dependent endocytosis behavior via interaction with β-arrestins (Gesty-Palmer and Luttrell 2011). PTH_1_R trafficking has notably been documented using synthetic agonist peptides conjugated to a chemical fluorophore (Ferrandon et al. 2009) or covalent linkage of a quantum dot fluorophore to the receptors (Zelman-Femiak et al. 2010). Furthermore, recent molecular pharmacologic studies showed that several hormones that bind secretin family GPCR hormones can be elongated at their C-terminus with a linker and transmembrane tether and still act as autocrine full agonists when coexpressed with a cognate receptor (Fortin et al. 2009, 2011) (this notably applied to PTH_1-34_; schematic representation, Fig. 1A). Altogether, these considerations led us to the hypothesis that it may be possible to prolong the C-terminus of PTH_1-34_ with a large biotechnological cargo that will be transported into cells via receptor-dependent endocytosis.

Several approaches supported a docking model for the nonapeptide bradykinin (BK) to its B_2_ receptor (B_2_R), a rhodopsin family GPCR (“class A”): the C-terminal part of the ligand was predicted to interact with the transmembrane domains, while the N-terminal end of BK rather binds to an extracellular loop (Leeb-Lundberg et al. 2005). Thus, it must be possible to extend the N-terminal sequence of BK to accommodate receptor-transported cargoes. Maximakinin, a natural sequence isolated from the skin of the amphibian *Bombina maxima*, the 19-mer DLPKINRKGPRPPGFSPFR, is composed of the full BK sequence at its C-terminal region with a hydrophilic 10-residue N-terminal extension. This peptide exhibits a 8- to 12-fold lesser potency versus BK at mammalian B_2_Rs (Bawolak et al. 2012). While N-terminal extensions of the BK sequence with various cargos generally lead to a severe loss of affinity at the B_2_R (Gera et al. 2012), we hypothesized that maximakinin (MK) includes a naturally selected sequence extension exploitable as a linker for the generation of agonist cargoes of this receptor.

With the objective of investigating the feasibility of large functional cargoes transported by receptors for peptide hormones, we fused both hormonal peptides, PTH_1-34_ and MK, to the enhanced green fluorescent protein (EGFP) to produce inherently fluorescent agonist probes for dissimilar GPCRs, the PTH_1_R, and B_2_R, respectively. The design of the fusion proteins took into consideration the orientation of each agonist peptide bound to its receptor, because the EGFP extension had to protrude out in the extracellular fluid (Fig. 1). The two recombinant proteins were found to be agonists of their respective receptors with nanomolar potencies and to be transported into the endocytic pathway of intact cells.

## Materials and Methods

### Construction of the PTH_1-34_-EGFP plasmid

The secreted form of EGFP (s-EGFP), containing the preproinsulin signal peptide, was previously reported (Jain et al. 2001) (gift from Dr. Paul Joyce, Concordia University, Montreal, Canada). The sequence encoding PTH_1-34_ was inserted following the signal peptide using oligonucleotide-directed site-specific mutagenesis, as described previously (Fortin et al. 2009). The composition of the resulting fusion protein expression vector (PTH_1-34_-EGFP) is detailed in Figure S1 and schematically represented in Figure 1B: the fluorescent protein is fused at the C-terminus of the hormonal peptide. The nucleotide sequences of all plasmids were confirmed by automated DNA sequencing; more specifically, the EGFP sequence with a “humanized” nucleotide usage and the modified N-terminal structure Met-Val was identified (Tsien 1998).

### Construction of the EGFP-MK plasmid

Two vectors containing the EGFP-MK fusion protein were successively produced: the first was designated s-EGFP-MK (Fig. S2). The preproinsulin signal, joining peptides and EGFP coding sequence of the original s-EGFP vector were retained, but the MK hormonal sequence was fused at the C-terminus of the coding sequence (schematic representation, Fig. 1C). The following oligonucleotide were annealed to generate double strand DNA coding for maximakinin (MK): 5′-G TAC AAG GAT TTG CCT AAG ATC AAC CGC AAA GGA CCA CGT CCA CCG GGG TTC TCC CCT TTT CGA TAA C-3′ and 5′-TCGAG TTA TCG AAA AGG GGA GAA CCC CGG TGG ACG TGG TCC TTT GCG GTT GAT CTT AGG CAA ATC CTT-3′. The dsDNA was then ligated into the BsrGI/XhoI digestion product of the s-EGFP vector using T4 ligase to generate the s-EGFP-MK vector. The recloned vector was validated by sequencing. The second vector, EGFP-MK, was produced by deleting the DNA sequences of the signal and joining peptides from the first one (boxed in Fig. S2) according to Hansson et al. (2008) and using the oligonucleotide polymerase chain reaction (PCR) primers 5′-CCC AAG CTT CTA GAC CAT GGT GAG CAA GGG CGA G-3′ (sense) and 5′-CTC GCC CTT GCT CAC CAT GGT CTA GAA GCT TGG G-3′ (antisense) followed by a DpnI digestion of the PCR product.

### Cell culture and production of biotechnological ligands

A subclone of HEK 293 cells, called HEK 293a, originally obtained from Sigma-Aldrich (St. Louis, MO) was used in all experiments. This cell type was grown in Dulbelcco's modified Eagle's medium (DMEM) supplemented with 10% fetal bovine serum, 1% l-glutamine, and 1% penicillin–streptomycin stock solutions (100×). The cells were used as recipients of the vectors s-EGFP and PTH_1-34_-EGFP. Seventy % confluent producer cells were transfected with a given vector using the ExGen reagent (Fermentas, Thermo Scientific, Ottawa, Canada) used as directed. Conditioned medium (CM) was collected after 24 h of culture for s-EGFP. In order to obtain consistent levels of PTH_1-34_-EGFP, a stable transfectant line has been developed by growing the cells for 1 month in the presence of geneticin (500 μg/mL; Invitrogen, Burlington, Canada) and sorting them based on fluorescence. CMs containing PTH_1-34_-EGFP were collected after 4 days of culture. Part of the CM was concentrated and desalted using the Amicon Ultra-15 Centrifugal Filter Units (Millipore, Billerica, MA; membrane cut-off 10 kDa).

Most experiments related to the BK B_2_R were based on the nonsecreted EGFP-MKconstruction (see Results). This cytosolic protein was produced as a lysate of HEK 293a cells previously (48 h) transfected before using a polyethyleneimine-based reagent as described (Morissette et al. 2008). The cells were then rinsed with phosphate buffered saline (PBS), left without supernatant, frozen for 2 h at −20°C, thawed, and scraped. The resulting suspension was centrifuged (15,000*g* 10 min) and the supernatant (the lysate) served as a concentrated stock of EGFP-MK for biochemical and pharmacological characterization. As a control, other cells were transfected with the EGFP-C3 vector (Clontech, Palo Alto, CA) and treated as described above for the preparation of a lysate. Cells were stimulated in their culture medium by lysates diluted at least 100-fold.

### Cell stimulation and analysis

Other recipient HEK 293a cells were grown and transiently transfected as described above with a vector coding for PTH_1_R (gift from Dr. T. J. Gardella, Massachusetts General Hospital) or for a fully functional rabbit BK B_2_R construction, myc-B_2_R (Bawolak et al. 2007). Some cells were optionally cotransfected with Cherry fluorescent protein (CherryFP), Rab5-GTP-locked-CherryFP or Rab7-CherryFP (given by Dr. M. J. Tremblay, Université Laval, Canada), or β-arrestin_1_-CherryFP (kind gift from Dr. J.-M. Beaulieu, Université Laval, Canada). The reporter plasmid that includes the CherryFP under the control of a multimeric (6 repeats) cyclic AMP responsive element (CRE-Cherry) has been described by Fortin et al. (2011). Stimulations for microscopic or cytofluorometric experiments were based on the CM or lysates for the GFP-based constructions. Cells were generally treated for 30 min (6 h for the CRE reporter gene) with stimulants (incubation carried out at 37°C in humidified atmosphere containing 5% CO_2_), rinsed three times with PBS, observed in microscopy for epifluorescence and photographed using an Olympus BX51 microscope (Center Valley, PA) coupled to a CoolSnap HQ digital camera (Photometrics, Tucson, AZ; filters for GFP: excitation 460–500 nm, emission 510–560 nm; for CherryFP: excitation 525–555 nm, emission 600–660 nm). The objective lens was generally the 100× oil UPlanApo (Olympus). Other cells treated in this manner were detached using the protease-free Cell Dissociation Buffer (Invitrogen), rapidly centrifuged (30 sec, 15,000*g*), resuspended in PBS, and analyzed using the BD SORP LSR II cell analyzer (BD Biosciences, Franklin Lakes, NJ; green or red fluorescence); results were analyzed using the BD FACS DIVA software.

Other transfected cells were detached using the Cell Dissociation Buffer, incubated in DMEM without serum at 37°C for 30 min under agitation in the presence of a stimulant, rapidly centrifuged (30 sec, 15,000*g*) and resuspended in PBS. Then, the fluorescence of the cell suspensions was assessed using the BD SORP LSR II cell analyzer for the uptake of a green fluorophore as a function of stimulation and transgene expression.

In addition to EGFP and derived fusion proteins, stimulants included synthetic PTH_1-34_ (Sigma-Aldrich) and forskolin (Calbiochem, La Jolla, CA), a direct adenylyl cyclase activator, BK (Bachem Biosciences, King of Prussia, PA) and anatibant (LF-16-0687; 1-[[2,4-dichloro-3-[(2,4-dimethylquinolin-8-yl)oxy]methyl]phenyl]sulfonyl]-N-[3-[[4-(aminoiminomethyl]-phenyl]carbonylamino]propyl]-2(S)-pyrrolidinecarboxamide, mesylate salt), a previously described nonpeptide B_2_R antagonist (Pruneau et al. 1999; gift from Laboratoires Fournier, Daix, France).

### Immunoblots

The identity and concentration of secreted or cytosolic GFP proteins was verified in immunoblotting experiments (10–15 μL of CM or 1 μL of cell lysate loaded per track; 9 or 10% SDS-polyacrylamide gels), performed as previously described (Bawolak et al. 2007) using the monoclonal anti-GFP antibodies JL8 (Clontech). The agonist action of PTH-related agonists was investigated using the expression of the transcription factor c-Fos, a distal signaling response to the stimulation of various receptor–ligand systems (Glauser and Schlegel 2007). Total HEK 293a cell extracts were immunoblotted to detect c-Fos expression using the K-25 rabbit polyclonal antibodies (Santa Cruz Biotechnology, Dallas, TX; dilution 1:50,000; 25 μg protein/track as measured by the Pierce BCA Protein Assay kit, Thermo Scientific). Equal track loading was further verified by migrating and transferring the same samples separately and immunoblotting for β-actin (monoclonal from Sigma-Aldrich; dilution 1:50,000).

### ELISA of GFP

A commercial GFP enzyme-linked immunosorbent assay (ELISA) kit (Cell Biolabs, Inc., San Diego, CA) was applied as directed to quantify the secreted s-EGFP and PTH_1-34_-EGFP in CMs (diluted 1:10 to 1:30,000, duplicate determinations) and EGFP-MK and EGFP in cell lysates (dilutions up to 1:150,000).

### Data analysis

Numerical values are reported as means ± SEM. Nonnormally distributed groups of values were analyzed using nonparametric analysis of variance (Kruskal–Wallis test) followed by Dunn's multiple comparison test. Normal sets of values were compared using analyses of variance (ANOVA) followed by Tukey–Kramer multiple comparison test or, for comparison with a common control value, Dunnett's test (InStat 3.05 computer program, GraphPad Software; San Diego, CA).

## Results

### Characterization of PTH_1-34_-EGFP

**Figure 2:**
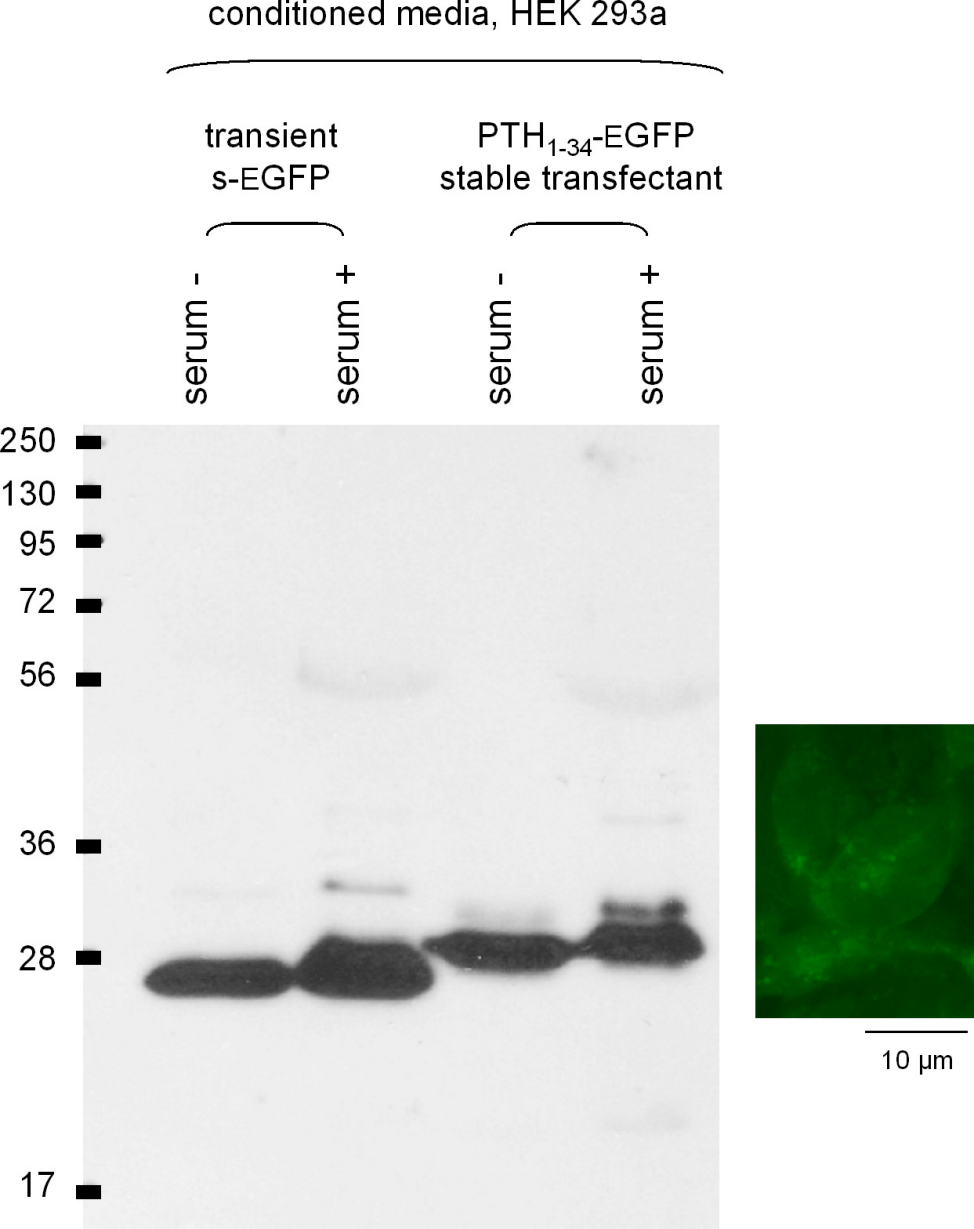
Immunoblots of the conditioned media (10 μL per track migrated in a 10% gel) of HEK 293a cells expressing either s-EGFP (transient transfection, 24-h collection) or PTH_1-34_-EGFP (stable transfectant, 96-h collection). The conditioned media were produced in the presence or absence of 10% fetal bovine serum, as indicated. Results representative of three experiments. Inset: Green epifluorescence of the stable transfectant cell line (400×).

The proteins secreted in the conditioned medium (CM) of transfected cells were characterized using immunoblots that exploited an anti-GFP monoclonal antibody (Fig. 2). HEK 293a transiently transfected with s-EGFP secreted the expected 27-kDa protein; the same cell type stably expressing the PTH_1-34_-EGFP vector secreted a relatively homogeneous protein heavier than GFP, consistent with the addition of the ∼4.1-kDa sequence. Collecting CM based on serum-free medium formulation decreased the yield of the fluorescent protein in each case (Fig. 2); therefore, the following experiments are based on the serum-containing CM. The cells that produced the PTH_1-34_-EGFP fusion protein contained granular cytosolic fluorescence (Fig. 2, inset), a distribution also shared with cells that transiently expressed s-EGFP (data not shown). Typical serum-containing CM collected from stable transfectant cells (4 days, PTH_1-34_-EGFP vector) or transiently transfected cells (s-EGFP, 24 h) contained an average of 1.5 ± 0.3 and 1.0 ± 0.1 nmol/L of GFP-related protein, respectively (ELISA for GFP, determination in three separately prepared CM for each). It was verified that the CM of untransfected HEK 293a cells contained no immunoreactive GFP.

**Figure 3:**
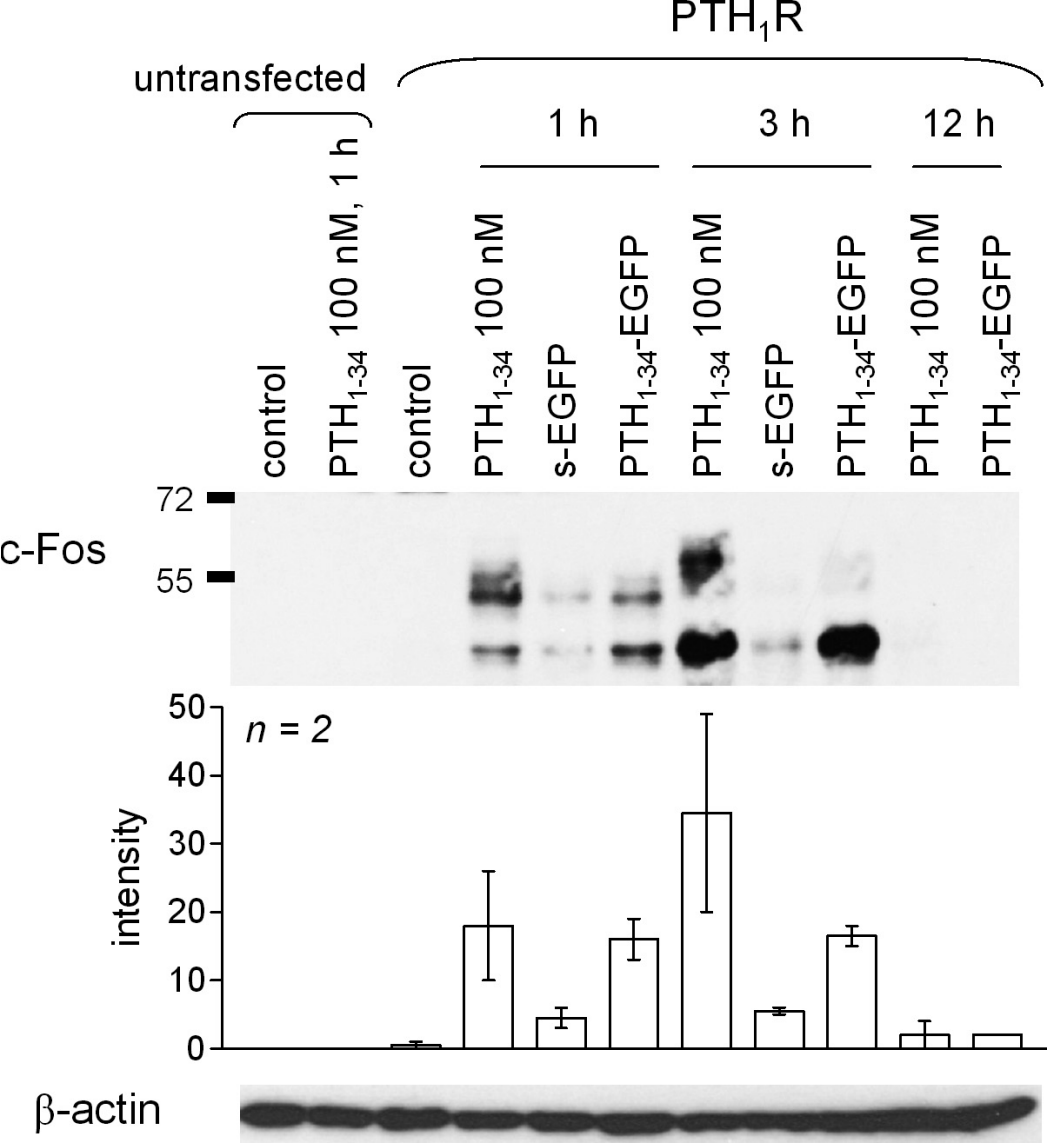
Induction of c-Fos in HEK 293a cells expressing or not PTH_1_R in response to treatments with conditioned media relevant for PTH_1-34_-EGFP or to PTH_1-34_. Results representative of two experiments. Histograms represent the intensity of c-Fos signal intensity under equal track loading conditions (25 μg/track). Track loading uniformity was further verified by β-actin immunoblotting.

We next verified whether the PTH_1-34_-EGFP construct retains agonist properties. Induction of the expression of the transcription factor c-Fos is a cell response distal to many receptor signaling systems; in HEK 293a cells that stably express PTH_1_R, synthetic PTH_1-34_ (100 nmol/L) activates it strongly 1 or 3 h after stimulation, but the effect vanished 12 h after stimulation (Fig. 3). Distinct apparent molecular weights of c-Fos are compatible with multiple phosphorylation events (Glauser and Schlegel 2007). The undiluted CM of cells producing PTH_1-34_-EGFP retained a fraction of the activity of the synthetic agonist, and also peaked at 3 h, while the CM containing s-EGFP was virtually inactive (Fig. 3).

**Figure 4:**
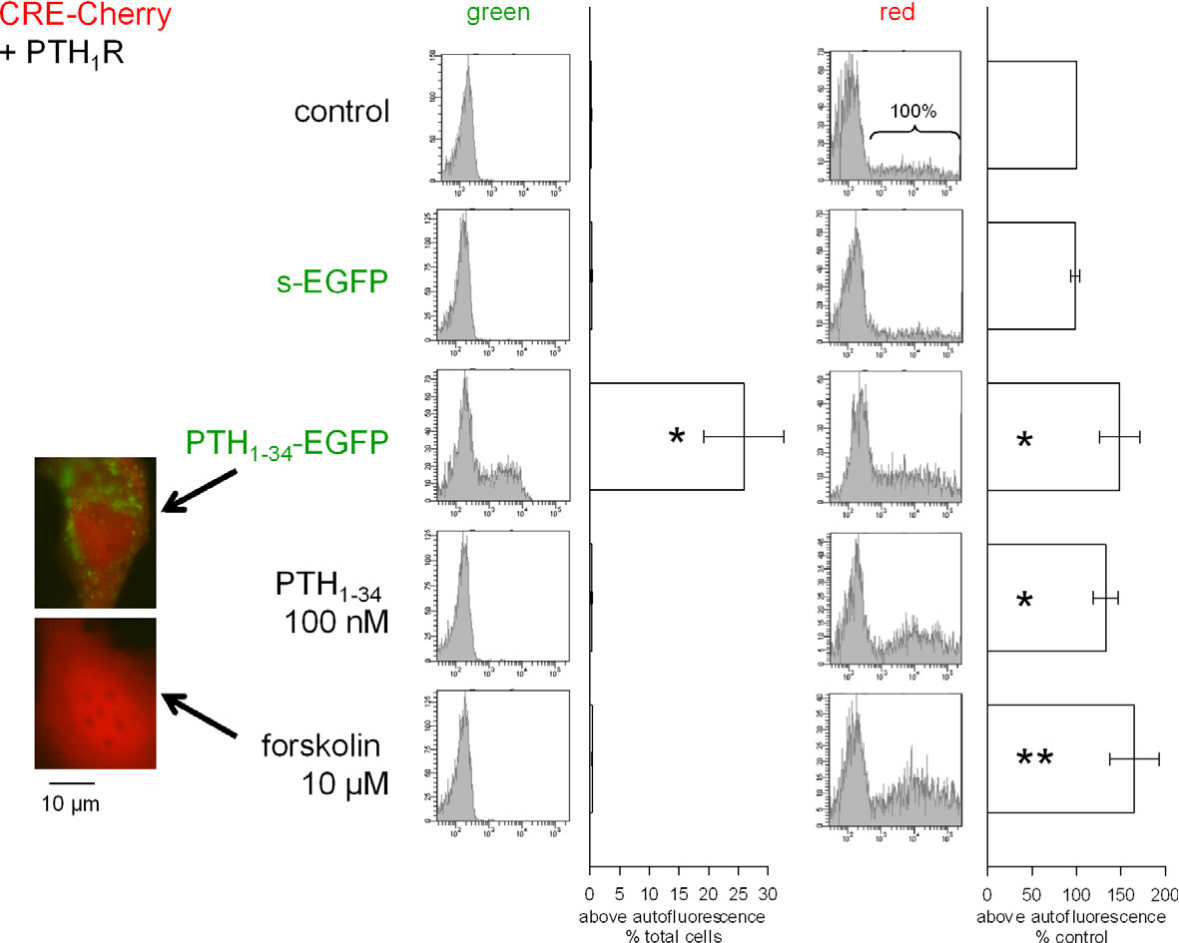
Expression of the Cherry fluorescent protein (CherryFP) in cells cotransfected with vectors coding for PTH_1_R and the Cherry reporter protein under control of cyclic AMP response elements (CRE). Cells were stimulated as indicated for 6 h. Histograms represent the cellular green fluorescence (center) or, on the right, the proportion of cells more fluorescent than the autofluorescence intensity normalized to the control group of each transfection (*n* = 5 or 6; distributions based on the counting of 10000 cells). The percentage of cells exhibiting a green fluorescence above autofluorescence in the cytofluorometry was significant only for cells stimulated PTH_1-34_-EGFP CM (*n* = 5 or 6; Kruskal–Wallis test *P* < 0.01; Dunn's multiple comparison test vs. controls: *P < 0.01). For the red fluorescence, the Kruskal–Wallis test indicated that the values were heterogeneous (*P* = 0.002). *P < 0.05 and **P < 0.01 versus control (Dunn's multiple comparison test). Insets: photomicrographs showing the uniform distribution of the CherryFP and the granular one for the EGFP fusion protein.

Receptor-mediated signaling was further evaluated using the CRE-Cherry reporter gene assay (CherryFP expressed under the control of a cAMP response element-based promoter). As PTH triggers the production of cyclic AMP (Kousteni and Bilezikian 2008), PTH_1-34_ (100 nM), PTH_1-34_-EGFP (CM), and forskolin (positive control) stimulated the expression of the red protein over the background in HEK 293a cells that expressed PTH_1_R (Fig. 4, right, cytofluorometry for the red fluorescence). s-EGFP-containing CM was not active in this respect. In this assay, a long stimulation period (6 h) allowed to monitor the accumulation of the PTH_1-34_-EGFP-associated fluorescence in multiple cytosolic granules in some cells and only this form of stimulation was associated with a green fluorescence intensity above the autofluorescence level (Fig. 4, inset and histograms at center). The intensity of the red and green fluorescence was highly correlated uniquely for cells treated with PTH_1-34_-EGFP (data not shown).

**Figure 5:**
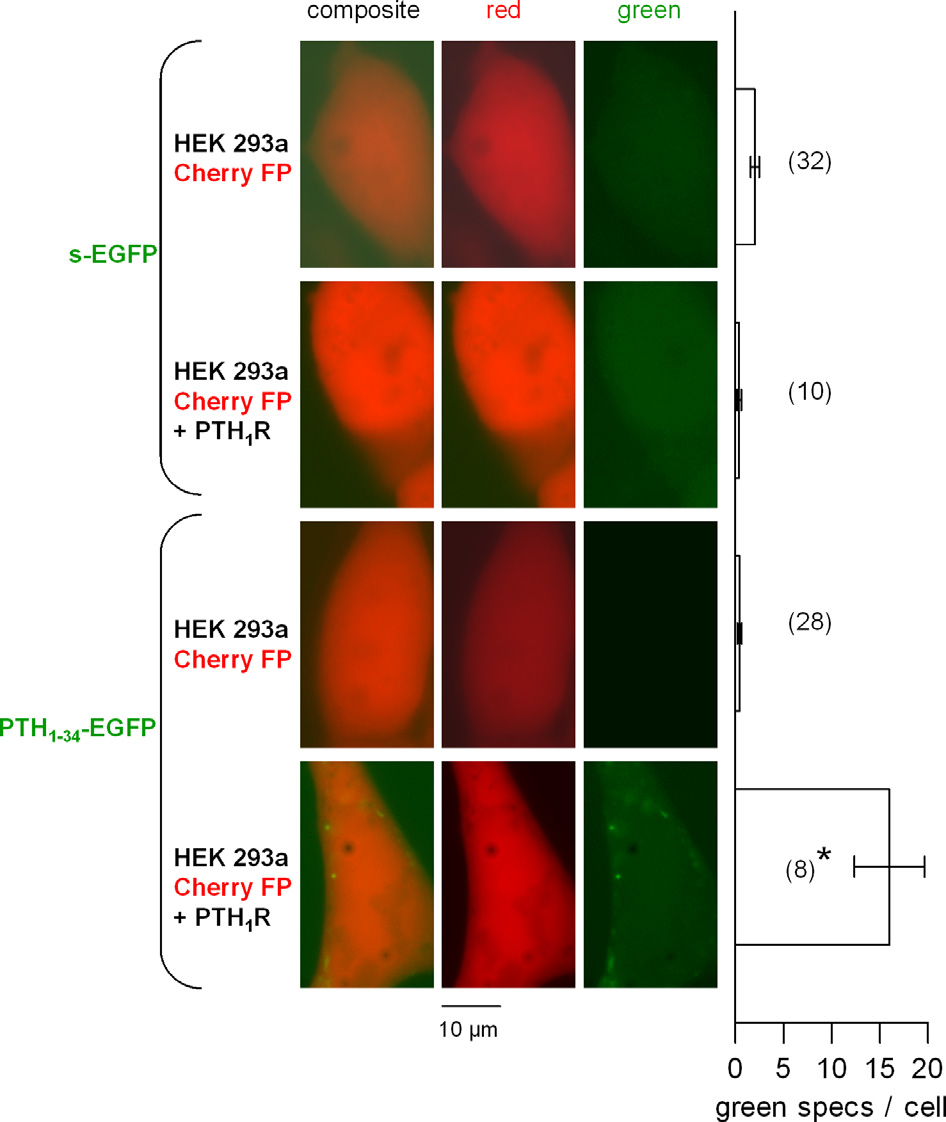
Endocytosis of PTH_1-34_-EGFP in HEK 293a cells that transiently expressed CherryFP and, optionally, PTH_1_R. s-EGFP was used as a control stimulus. The undiluted conditioned media were transferred for a 30-min incubation period before rinsing and observation. Original magnification 1000×. Right: Number of green intracellular condensed structures (“specs”) per cell ± SEM. Numbers close to bars indicate the number of evaluated HEK 293a cells. The Kruskal–Wallis test indicated that the values were heterogenous (*P* < 0.001). The effect of receptor presence was evaluated using Dunn's multiple comparison test for each construction. *P < 0.001.

**Figure 6:**
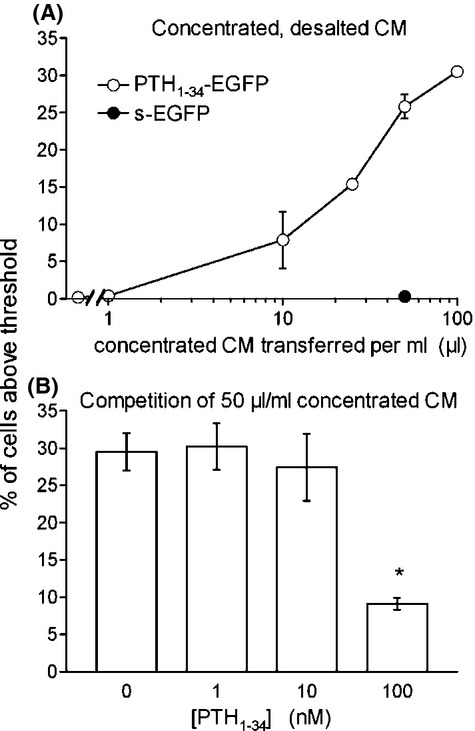
(A) Proportion of PTH_1_R-expressing HEK 293a cells specifically stained with a concentrated and desalted preparation of PTH_1-34_-EGFP as a function of the concentration (cytofluorometry gated as in Figure S3). (B) Competition of PTH_1-34_-EGFP uptake by cotreatment with unlabeled PTH_1-34_ in receptor-expressing cells. ANOVA indicated that the values were heterogeneous (*P* < 0.001); *P < 0.01 versus control (Dunnett's). Number of replicates is 1–4 in A, 5 or 6 in B.

We further studied agonist-induced PTH_1_R intracellular endocytosis. The CM were transferred for a 30 min incubation period (37°C) to recipient HEK 293a cells that transiently expressed CherryFP and, optionally, PTH_1_R, the former transgene being used to identify transfected cells (Fig. 5). Coarse and intensely green fluorescent granules were observed following incubation of cells (30 min, 37°C) with the CM containing the PTH_1-34_ fusion protein only when the receptor was present; the CM containing s-EGFP did not significantly mark the cells in the presence or absence of the receptors. A similar protocol, based on the fluorometry of cells expressing or not PTH_1_R and sequentially detached and stimulated, also showed specific staining (above autofluorescence) only with the PTH_1-34_-EGFP CM if the receptor was present; all other conditions were negative (staining with s-EGFP or in absence of receptors, Fig. S3). A ∼50-fold concentrated and desalted PTH_1-34_-EGFP CM, obtained using centrifugal filter units, stained PTH_1_R-expressing HEK 293a cells in a concentration-dependent manner. In contrast, concentrated CM containing s-EGFP was not active to stain PTH_1_R containing cells (Fig. 6A, cytofluorometry gated as in Fig. S3). Following treatment with a fixed proportion of concentrated CM containing PTH_1-34_-EGFP, the uptake of the fusion protein by receptor-expressing cells was prevented by unlabeled PTH_1-34_ at 100 nM only (Fig. 6B).

**Figure 7:**
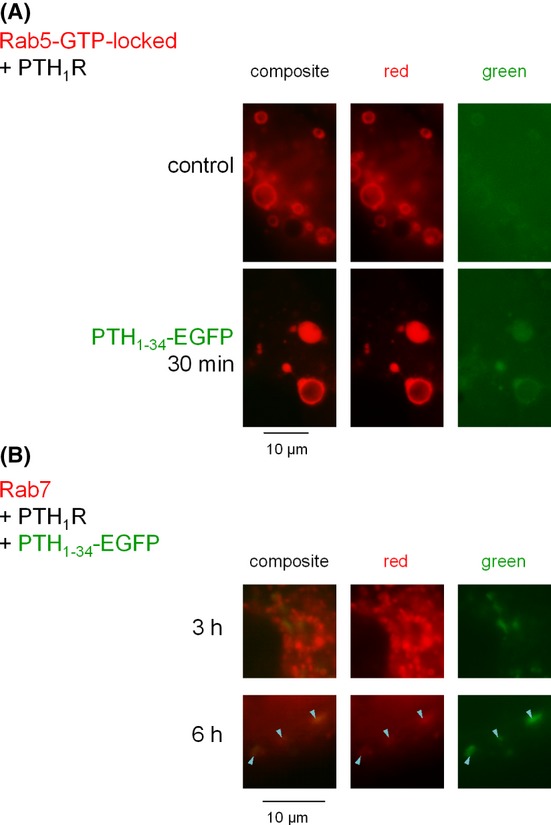
Colocalization studies in HEK 293a cells coexpressing PTH_1_R and CherryFP-conjugated Rab construction. (A) GTP-locked Rab5 colocalization. Cells were stimulated with the CM containing PTH_1-34_-EGFP for 30 min; control cells were used to evaluate the green autofluorescence and Rab5 distribution under the same conditions. (B) Rab7 colocalization studies for longer incubation periods (as indicated). The colocalization is very rare at 3 h, but significant at 6 h (arrowheads). Epifluorescence, original magnification: 1000×.

A mutant Rab5 construction exhibiting constitutive activity (GTP-locked) is known to cause the formation of giant endosomes where the internalized cargo stops its progression (Stenmark et al. 1994). A CherryFP-conjugated GTP-locked Rab5 was present at the surface of such giant vacuoles that became decorated with green fluorescence when treated with PTH_1-34_-EGFP CM for 30 min (Fig. 7A), supporting that the fusion protein follows a classical endocytic route. The small G protein Rab7 is rather associated with late endosomes and lysosomes (Grosshans et al. 2006). Colocalization of Rab7-CherryFP with PTH_1-34_-EGFP was not observed after 3 h of treatment in cells that expressed PTH_1_R, but was significant after 6 h (Figure 7B), suggesting a slow progression of the agonist fusion protein toward lysosomes.

### Characterization of EGFP-MK

**Figure 8:**
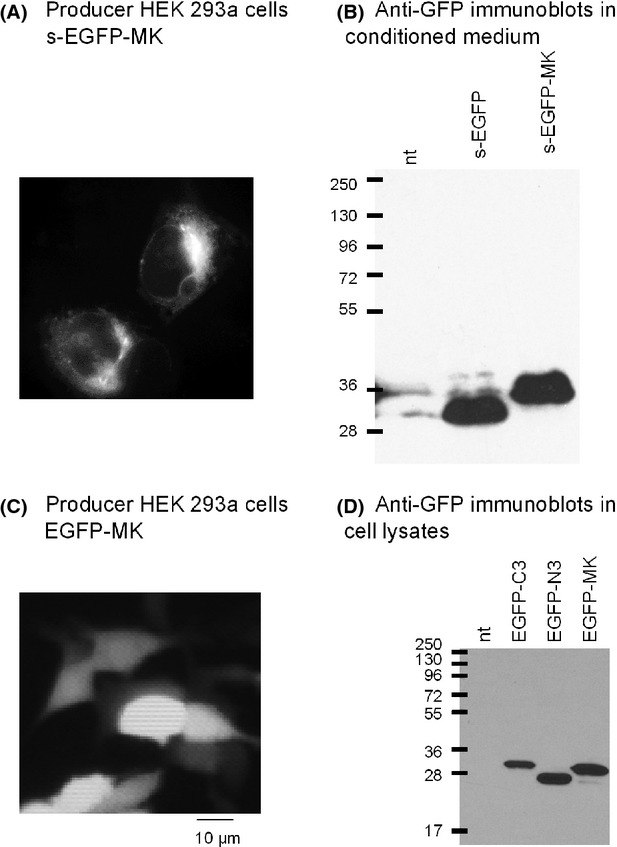
Production of the proteins coded in the s-EGFP-MK and EGFP-MK vectors. HEK 293a cells transiently transfected with the first vector exhibit a granular fluorescence distribution, whereas EGFP-MK produces a uniform labeling of all cell water (A, C). The proteins secreted in the conditioned medium of cells expressing s-EGFP-MK or recovered in the lysate of cells expressing EGFP-MK (immunoblots exploiting an anti-GFP monoclonal antibody (B, D); representative of at least two replicates) are heavier than the GFP produced by cells expressing secretory EGFP, but slightly lighter than that encoded by the C-terminally extended EGFP-C3.

HEK 293a cells were transiently transfected with the s-EGFP-MK vector; after 24 h, they exhibited a granular fluorescence distribution (Fig. 8A), consistent with the fluorescent protein being produced in the secretory pathway. The protein secreted during 96 h in the conditioned medium (CM) of transfected cells (immunoblot exploiting an anti-GFP monoclonal antibody, Fig. 8B) is heavier than the GFP secreted by cells expressing secretory EGFP, consistent with the addition of the ∼2.2-kDa MK sequence. However, morphological and pharmacological evaluation of the supernatants containing s-EGFP-MK did not convincingly show the presence of a BK B_2_R ligand (data not shown). Therefore, the exploitation of the nonsecretory EGFP-MK was attempted. Cells that expressed this construction were uniformly labeled in all their water content (Fig. 8C). Such cells, rinsed with PBS, frozen and thawed, were the source of a cytosolic lysate that contained an apparently pure protein reactive with the anti-GFP antibody, of a molecular weight just slightly inferior to the 34-kDa EGFP coded by the Clontech pEGFP-C3 vector prepared in the same manner (immunoblot, Fig. 8D). EGFP-C3 has an arbitrary 26-residue C-terminal extension due to the translation of a multiple cloning site, but not the product of the alternate EGFP-N3 vector that represents the 27-kDa GFP and that is also shown in Figure 8D. The fact that both C-terminally extended sequences EGFP-C3 and EGFP-MK exhibit the correct molecular weight with little degradation is consistent with the high stability of GFP in mammalian cells (Corish and Tyler-Smith 1999). The lysates were standardized and the concentration of the proteins was evaluated using the anti-GFP ELISA. Two different EGFP-MK lysates contained 0.83 and 2.5 μM immunoreactive GFP (average 1.67 μM); one EGFP-C3 lysate, based on a different expression system, contained 30 nmol/L immunoreactive GFP. In subsequent experiments, the EGFP-C3 lysate was used as a control for any possible pharmacologically active contaminant from producer cell lysate (designated as HEK 293a lysate), while its EGFP content did not compare well to the concentration of the fusion protein EGFP-MK.

**Figure 9:**
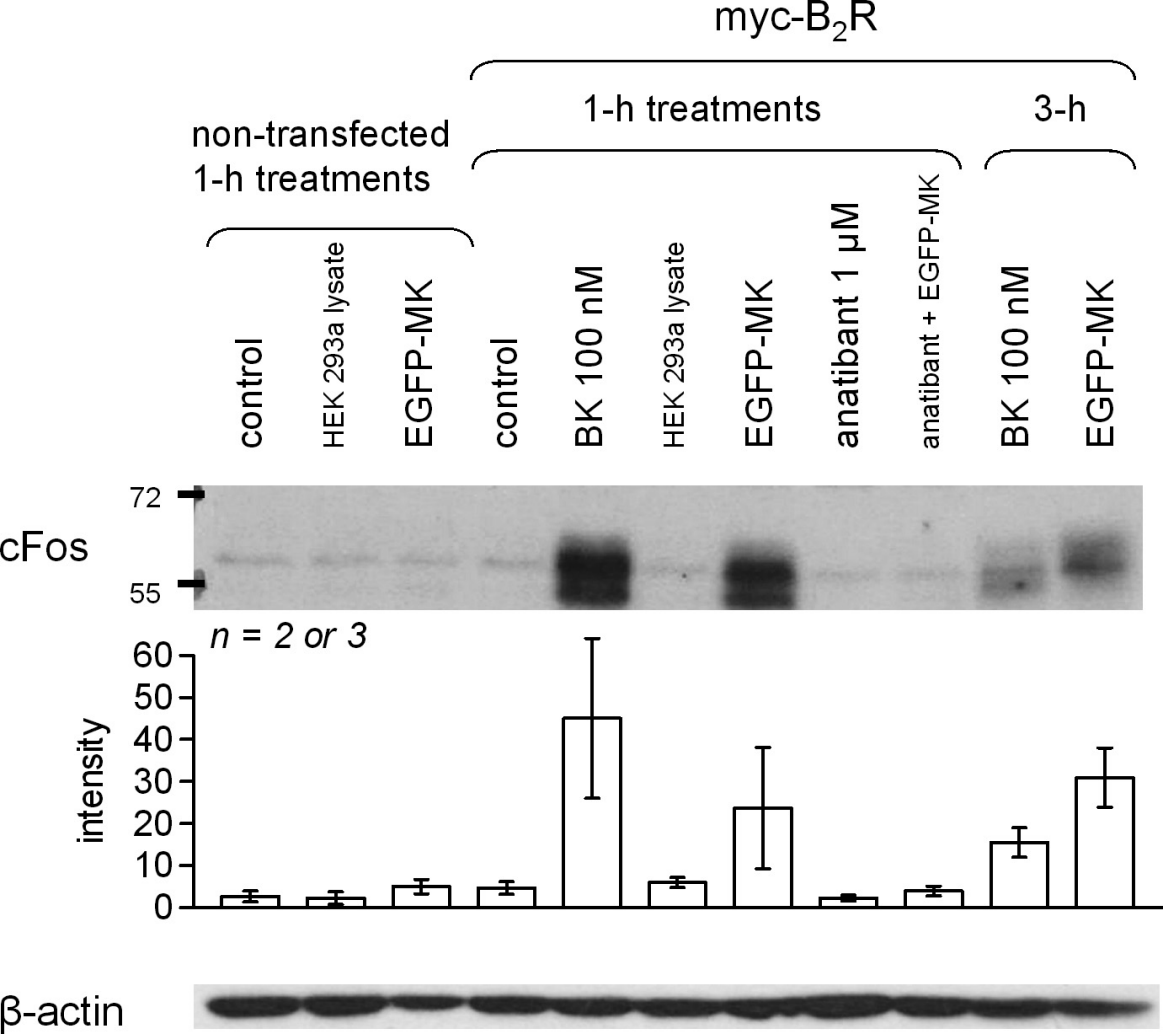
Induction of c-Fos in HEK 293a cells expressing or not myc-B_2_R in response to treatments with diluted lysate containing EGFP-MK (final concentration 12.6 nmol/L), with similarly diluted HEK 293a cell lysate (from cells transfected with the EGFP-C3 vector) or authentic BK. Results are representative of two or three experiments, depending on conditions. Presentation as in Figure 3.

As for the PTH_1-34_-EGFP system, we verified that the EGFP-MK construct retained agonist properties using the c-Fos induction assay in HEK 293a that optionally expressed the recombinant myc-B_2_R (Fig. 9). As previously reported, authentic BK (100 nmol/L) induced c-Fos in receptor-expressing cells, and 1 h of treatment leads to the strongest response, while it subsided 3-h poststimulation. EGFP-MK (final concentration of 12.6 nmol/L in this set of experiments) had sustained activity after 1 or 3 h of treatment (Fig. 9), with higher average molecular weight at 3 h compatible with hyperphosphorylation and nuclear translocation (Glauser and Schlegel 2007). The B_2_R antagonist anatibant abated the acute effect of EGFP-MK. Lysates of control HEK 293a cells were uniformly inactive.

**Figure 10:**
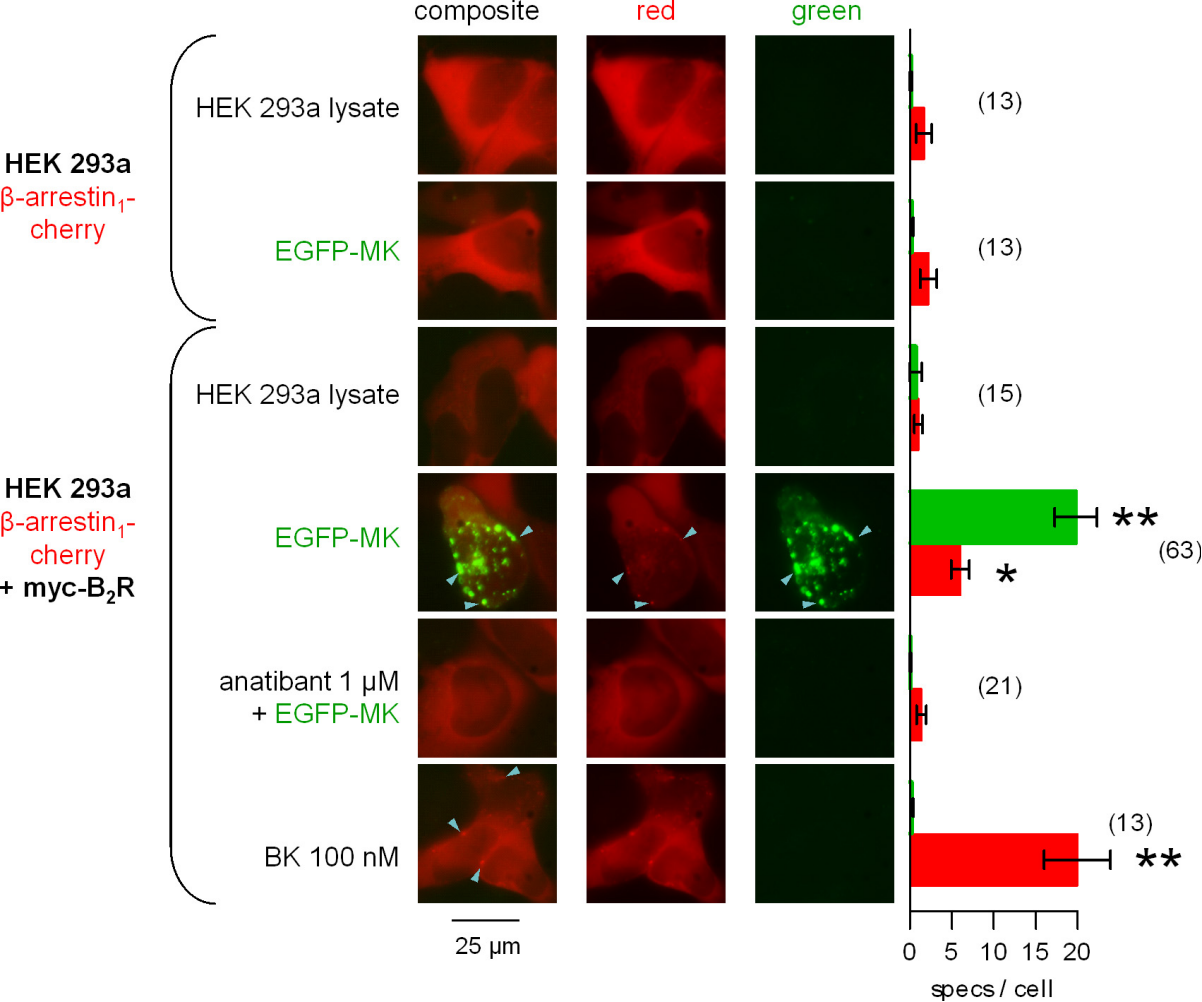
Epifluorescence microscopy studies in cells coexpressing β-arrestin_1_-Cherry and, optionally, myc-B_2_R and stimulated for 30 min with the indicated agent. EGFP-MK, recovered from cell lysate, was diluted to a final concentration of 4.2 nmol/L for EGFP-MK (estimate based on an ELISA for GFP). Control HEK 293a cell lysate, used in equal proportion, was from cells transfected with the EGFP-C3 vector. Original magnification: 1000×. Only cells expressing the myc-B_2_R and stimulated with EGFP-MK exhibited green endocytic structures, and some were colocalized with condensed arrestin (arrowheads). BK stimulation also produced arrestin condensation, but without green labeling (arrowhead). Right: Number of intracellular condensed structures (“specs”) per cell for each of the green and red signals. Numbers between parentheses close to bars indicate the number of evaluated HEK 293a cells. The Kruskal–Wallis test indicated that the values were heterogenous for both the red and green signals (*P* < 0.001). The effect of stimulant treatment relative to control cells (receptor-expressing cells stimulated with EGFP, 3rd row) was evaluated using Dunn's multiple comparison test for each color. *P < 0.05; **P < 0.001.

The lysates, collected after 48 h of culture, were transferred for a 30 min incubation period (37°C) to recipient HEK 293a cells that transiently expressed β-arrestin_1_-Cherry and, optionally, myc-B_2_R (Fig. 10). Coarse and intensely fluorescent granules were observed for the MK fusion protein only in cells that expressed the receptor. A strong stimulation of cells with nonfluorescent BK (100 nM) condensed the cytosolic red fluorescence of β-arrestin_1_-Cherry into endosomal structures (arrowheads), and those were colocalized with a fluorescent BK analog in a previous study (Gera et al. 2011). The control lysate had no effect, notably on the condensation of the arrestin fusion protein. The EGFP-MK lysate somewhat increased the number of red particles per cell (Fig. 10), and the green structures were occasionally colocalized with red ones (arrowheads). The nonpeptide B_2_R antagonist anatibant abated the uptake of EGFP-MK in cells that expressed myc-B_2_R (Fig. 10).

**Figure 11:**
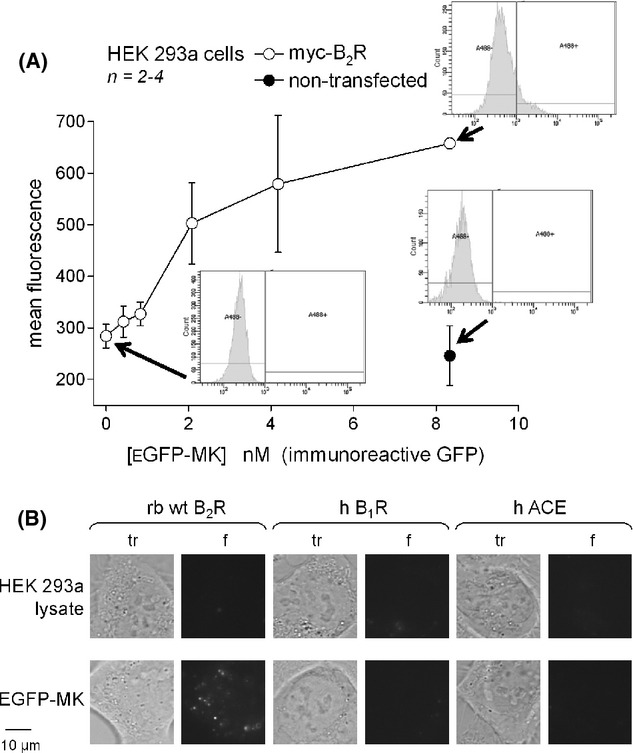
(A) Cytofluorometry of HEK 293a cells that optionally expressed myc-B_2_R, sequentially detached and stained for 30 min (37°C) with EGFP-MK recovered from the cell lysate of producer cells (concentration indicated as nmol/L of immunoreactive GFP). (B) Selectivity of HEK 293a cell labeling by EGFP-MK (4.2 nmol/L, 30 min, 37°C; control HEK 293a lysate contained 0.15 nmol/L EGFP) as a function of the expressed transgene (rabbit wild-type B_2_R, human kinin B_1_ receptor, human angiotensin-converting enzyme). Matched transmission (tr) and green fluorescence (f) fields are shown side by side (1000×).

A quantitative cytofluorometry approach confirmed that only recipient cells that expressed the receptor (myc-B_2_R) and were exposed to the EGFP-MK lysate for 30 min exhibited some specific green fluorescence above the autofluorescence level (Fig. 11A). EGFP-MK had a nanomolar potency at least as good as authentic MK in systems based on B_2_Rs (Bawolak et al. 2012); this conclusion is based on the calculated EGFP-MK concentration derived from its immunoreactivity in the GFP ELISA. The labeling intensity was not strong, under the relatively unfavorable circumstance where the fluorescence in individual cells is granular, not well distributed. The specificity of the binding of EGFP-MK to alternate molecules belonging to the kallikrein-kinin system was verified (Fig. 11B). HEK 293a cells transiently expressing human recombinant B_1_ receptors for kinins (hB_1_R-FLAG vector reported by Morissette et al. 2008) or human recombinant angiotensin-converting enzyme (ACE; vector peACE, Wei et al. 1991) failed to capture EGFP-MK; the rabbit wild type B_2_R was the positive control in these experiments (Fig. 11B). The expression of the B_1_ receptor and ACE transgenes at the surface of HEK 293a transfected as shown here is well documented using the radioligands [^3^H]Lys-des-Arg^9^-BK and [^3^H]enalaprilat, respectively (Morissette et al. 2008; Gera et al. 2011). ACE is not a GPCR, but an ectopeptidase that constitutes a specific binding site for BK and that is labeled in such cells by the derivative carboxyfluorescein-ε-aminocaproyl-BK (Gera et al. 2011).

**Figure 12:**
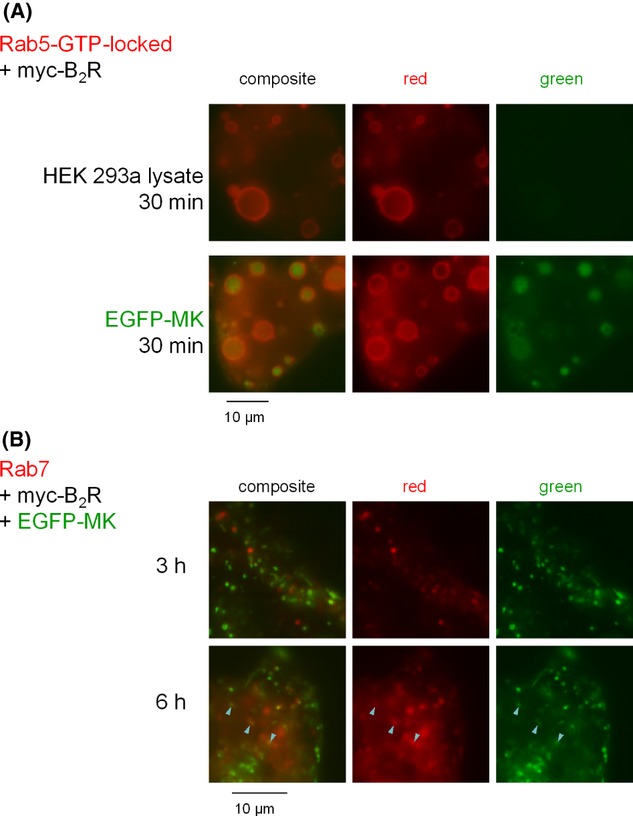
Colocalization studies in HEK 293a cells expressing myc-B_2_R and CherryFP-conjugated Rab constructions. (A) GTP-locked Rab5 colocalization. Cells were stimulated as in Figure 11B with the lysate of cells transiently expressing EGFP-C3 (control) or EGFP-MK. (B) Rab7 colocalization studies for longer incubation periods (as indicated). The colocalization is very rare at 3 h, but significant at 6 h (arrowheads indicate some examples). Epifluorescence, original magnification: 1000×.

As for PTH_1-34_-EGFP, cells that expressed both Rab5-GTP-locked and the appropriate receptor and further stimulated with EGFP-MK trapped this construction in the giant endosomes that are recognizable by their red fluorescent lining (Fig. 12A). In cells stimulated with EGFP-MK for longer periods (3–6 h), the fusion protein was not released and a proportion of vacuoles containing it were apparently labeled with Rab7 at time 6 h (Fig. 12B), suggesting a progression in the late endosome/lysosome tract.

Additional experiments, reported in the Figure S4, showed the lack of nonspecific uptake of each EGFP fusion protein by the noncorresponding receptor stimulated with a peptide agonist. Thus, there is no uptake of PTH_1-34_-EGFP in cells that express the B_2_R and that are stimulated with BK; there is no uptake of EGFP-MK when PTH_1_R is stimulated with PTH_1-34_. These results confirm the specificity of the EGFP ligands and exclude certain modes of uptake that could be somehow facilitated by cell stimulation, like pinocytosis (fluid phase endocytosis), that mediated by cell-penetrating peptides (the C-terminal sequence of EGFP-MK containing several Arg residues) or that caused by the hypothetical residual presence of transfection reagents.

## Discussion

Because the secretin receptor family is tolerant to variations in the C-terminal structure of peptide ligands (Fortin et al. 2009), we hypothesized that a protein that included PTH_1-34_ at its N-terminus would stimulate and be transported into cells by the activated PTH_1_R. This was amply confirmed using the ∼31-kDa fusion protein PTH_1-34_-EGFP, active in signal transduction experiments (Figs. 3, 4) and taken up into endosomal structures only in cells that expressed the receptor (Figs. 5, 7, Fig. S3). Supporting the specificity of PTH_1-34_-EGFP actions, the parent protein s-EGFP was uniformly inactive in all of the assessed outcomes. These results were obtained despite technical limitations due to the expression system, where only low nanomolar concentrations of these secreted fusion proteins were recovered in the CM of producing cells. Indeed, even using concentrated and desalted CM, the maximum uptake of the fusion protein PTH_1-34_-EGFP was not reached (Fig. 6A). Based on the competition of its cellular uptake by the synthetic peptide PTH_1-34_ (Fig. 6B), the affinity of PTH_1-34_-EGFP for PTH_1_R appears to be of the same order of magnitude than that of PTH_1-34_, a stimulant of various bioassays with an EC_50_ between 10 and 100 nmol/L (Nickols 1985). There may be a discrepancy between the potency of authentic PTH_1-34_, used at 100 nmol/L, and that of PTH_1-34_-EGFP (approximately 1.5 nmol/L) when considering their approximate equal effect on long term (1–6 h) functional responses, c-Fos induction, and CRE-Cherry expression (Figs. 3, 4). At least two factors can explain the apparent discrepancy: PTH_1-34_ is rapidly degraded by serum (Seibel et al. 1996), but apparently not PTH_1-34_-EGFP as the relatively homogeneous fusion protein is present in the serum-containing CM (Fig. 2). PTH analogs and fragments widely differ in their PTH_1_R cycling kinetics and differential (biased) signaling (Gesty-Palmer and Luttrell 2011; Vilardaga et al. 2012). Nevertheless, PTH_1-34_-EGFP effects are specific, always dependent on the presence of the receptor, and the fusion protein is a fluorescent agonist that can be exploited in cell imaging experiments, as shown in HEK 293a cells (Figs. 4, 5, 7).

High molecular weight ligands of some rhodopsin family GPCRs have been produced, for instance fusion proteins composed of substance P or the chemokine CCL19 and immunoglobulin sequences (Otero et al. 2006; Rizk et al. 2009). Current models of BK docking to its B_2_R suggest that the C-terminus of the ligand plunges into the central receptor cavity and that the N-terminus is closer to the extracellular fluid. This is consistent with the fact that MK, with its rather large N-terminal prolongation versus BK, binds to and stimulates the B_2_R with only a moderate loss of affinity (∼1-log unit, Bawolak et al. 2012). This loss is much less drastic than the ∼3-log ones in BK analogs prolonged with chemical fluorophores at the N-terminus (Gera et al. 2012). Present results support that the hydrophilic extension of the BK sequence present in MK is a viable spacer between the large and compactly organized cargo such as GFP. Estimates of EGFP-MK potency, based on the cytofluorometric assay, show that the fusion protein has an affinity at least as good, or possibly better than that of MK for the B_2_R if the ELISA for GFP does not underestimate EGFP-MK concentration due to the sequence extension. GFP is a stable globulin in mammalian cells (Corish and Tyler-Smith 1999) and EGFP-MK seems to be equally protected from degradation as the cell lysate of producer cells contained a pharmacologically active and immunoreactive fusion protein of the right molecular mass.

EGFP-MK has no significant effect in cells that don't express B_2_Rs (Figs. 9, 10, 11) and, in the presence of the receptor, its effects are abolished by cotreatment with the antagonist anatibant (Figs. 9, 10). These considerations sufficiently support the specificity of the probe. Further, current results support that EGFP-MK is highly selective for the BK B_2_R as the fusion protein was not taken up by cells that expressed the kinin B_1_ receptor (selective for kinin metabolites from which the C-terminal arginine residue has been removed; Leeb-Lundberg et al. 2005) or the kinin-destroying peptidase ACE (Fig. 11B). Thus, EGFP-MK has a distinctive selectivity over carboxyfluorescein-ε-aminocaproyl-BK, a peptide that labels both B_2_Rs and ACE in the same concentration range (Gera et al. 2011). The reason for this is possibly that EGFP-MK is excluded from the catalytic sites of ACE, being a fairly large protein. The resistance of EGFP-MK to ACE (inferred from Fig. 11B) and the very rapid degradation of BK both in serum-containing culture medium and endosomes (Bawolak et al. 2012) may account for a certain potency discrepancy in the c-Fos induction assay (Fig. 9) where the 12.6 nmol/L EGFP-MK level has an effect approximately equal to that of 100 nmol/L BK after 1 h of stimulation. Further, MK has a much more prolonged effect than BK in this assay, possibly due to resistance to endosomal degradation (Bawolak et al. 2012), and present results suggest that EGFP-MK may have inherited this prolonged action (Fig. 9, effect at 3 h stronger than that of BK).

Both GFP-containing agonist probes are internalized into Rab5 positive early endosomes (Figs. 7, 12), typical of the agonists of the B_2_R and PTH_1_R. This transport is preceded by the phosphorylation of each receptor type and combination with arrestins (Leeb-Lundberg et al. 2005; Cupp et al. 2013) which could be demonstrated using significant endosomal colocalization of EGFP-MK with β-arrestin_1_-Cherry in cells that expressed myc-B_2_R (Fig. 10). However, the same could not be shown in the PTH_1-34_-EGFP/PTH_1_R system (data not shown), probably due to the low concentration of the agonist in this case (concentrated PTH forms are active in this respect in our hands, data not shown). While both PTH_1_R and B_2_R are essentially recycled back to the plasma membrane after agonist-induced endocytosis (Leeb-Lundberg et al. 2005; Zelman-Femiak et al. 2010), the progressive colocalization with Rab7 suggests that both EGFP fusion proteins progress toward lysosomes. In the maximal period of observation (6 h), the associated green fluorescence was not released or appear in the cytosol.

While both the activated PTH_1_R and B_2_R were previously shown to transport large receptor-bound cargoes, such as Qdot nanomaterials (Zelman-Femiak et al. 2010; Bawolak et al. 2011), the constructs PTH_1-34_-EGFP and EGFP-MK represent *bona fide* agonists that support the feasibility of a ligand-centered strategy to transport protein cargoes inside cells using GPCRs. Indeed, the typical B_2_R agonist BK, a 1-kDa nonapeptide, and PTH_1-34_ (4 kDa) are much smaller than the novel agonist fusion proteins that we have produced and characterized.

## Abbreviations

ACE: angiotensin-converting enzyme
B_2_R: B_2_ receptor
BK: bradykinin
cherryFP: cherry fluorescent protein
CM: conditioned medium
CRE: cyclic AMP response element
EGFP: enhanced green fluorescent protein
EGFP-MK: EGFP fused to maximakinin
ELISA: enzyme-linked immunosorbent assay
GFP: green fluorescent protein
GPCR: G protein-coupled receptor
GRK: GPCR kinase
MK: maximakinin
myc-B_2_R: myc-tagged rabbit B_2_ receptor
PBS: phosphate buffered saline
PTH: parathyroid hormone
PTH_1-34_-EGFP: PTH 1-34 fragment fused to EGFP
PTH_1_R: parathyroid hormone receptor-1
s-EGFP: secretory EGFP

## Supporting Information

**Figure S1.** Partial coding sequence of the PTH_1-34_-EGFP vector. See text for description. **Figure S2.** Partial coding sequence of the s-EGFP-MK and the EGFP-MK vectors, the second being obtained following the deletion of the boxed sequence (signal peptide + joining peptide). See text for description. **Figure S3.** Cytofluorometry of HEK 293a cells that optionally expressed PTH_1_R (3 conditions at bottom), sequentially detached and stained for 30 min (37°C) with the undiluted conditioned medium of other cells producing s-EGFP or PTH_1-34_-EGFP, as indicated. Left: distributions based on the counting of 10,000 cells. A threshold of autofluorescence was defined using control cells with no fluorophore. It was surpassed only under one set of experimental conditions (arrow). Right: proportion of cells above the threshold under each experimental condition in three separate experiments (means ± SEM). ANOVA indicated that the values were heterogeneous (*P* < 0.02). **P* < 0.05 versus controls with or without receptors (Tukey–Kramer multiple comparison test). s-EGFP did not significantly stain cells. **Figure S4.** Lack of stimulated uptake of fluorescent probes by cells expressing the non-corresponding receptor. HEK 293a cells expressing the indicated receptor were incubated for 30 min with one of the EGFP fusion protein and optionally stimulated with a non-fluorescent peptide, as indicated.
