# Supplementary material for: Green fluorescent protein fused to peptide agonists of two dissimilar G protein-coupled receptors: novel ligands of the bradykinin B2 (rhodopsin family) receptor and parathyroid hormone PTH1 (secretin family) receptor
Source: Pharmacol Res Perspect. 2013 Oct 4;1(1):e00004. doi: 10.1002/prp2.4 (PMC4184569; doi:10.1002/prp2.4)
Supplement: Supplementary file 1 — Figure S1. Partial coding sequence of the PTH1-34-EGFP vector. See text for description. Figure S2. Partial coding sequence of the s-EGFP-MK and the EGFP-MK vectors, the second being obtained following the deletion of the boxed sequence (signal peptide + joining peptide). See text for description. Figure S3. Cytofluorometry of HEK 293a cells that optionally expressed PTH1R (3 conditions at bottom), sequentially detached and stained for 30 min (37°C) with the undiluted conditioned medium of other cells producing s-EGFP or PTH1-34-EGFP, as indicated. Left: distributions based on the counting of 10,000 cells. A threshold of autofluorescence was defined using control cells with no fluorophore. It was surpassed only under one set of experimental conditions (arrow). Right: proportion of cells above the threshold under each experimental condition in three separate experiments (means ± SEM). ANOVA indicated that the values were heterogeneous (P < 0.02). *P < 0.05 versus controls with or without receptors (Tukey–Kramer multiple comparison test). s-EGFP did not significantly stain cells. Figure S4. Lack of stimulated uptake of fluorescent probes by cells expressing the non-corresponding receptor. HEK 293a cells expressing the indicated receptor were incubated for 30 min with one of the EGFP fusion protein and optionally stimulated with a non-fluorescent peptide, as indicated. [file prp20001-e00004-sd1.pdf]

PTH$_{1-34}$-EGFP vector

ACC*ATGGCCCTGTGGATGCGCCTCCTGCCCCTGCTG*
MetAlaLeuTrpMetArgLeuLeuProLeuLeu
preproinsulin signal peptide

GCGCTGCTGGCCCTCTGGGGACCTGACCCAGCCGCA
AlaLeuLeuAlaLeuTrpGlyProAspProAlaAla

*GCG**TCTGTGAGTGAAATACAGCTTATGCATAACCTG***
*Ala**SerValSerGluIleGlnLeuMetHisAsnLeu***
Signal peptidase complex
PTH$_{1-34}$

GGAAAACATCTGAACTCGATGGAGAGAGTAGAATGGCTG
GlyLysHisLeuAsnSerMetGluArgValGluTrpLeu

CGTAAGAAGCTGCAGGATGTGCACAATTTT
ArgLysLysLeuGlnAspValHisAsnPhe

GATCCACCGGTCGCCACCATGGTG...AAGTAA
AspProProValAlaThrMetVal...Lys Stop
joining peptide EGFP →

Supplementary Figure 1. Partial coding sequence of the PTH$_{1-34}$-EGFP vector. See text for description.

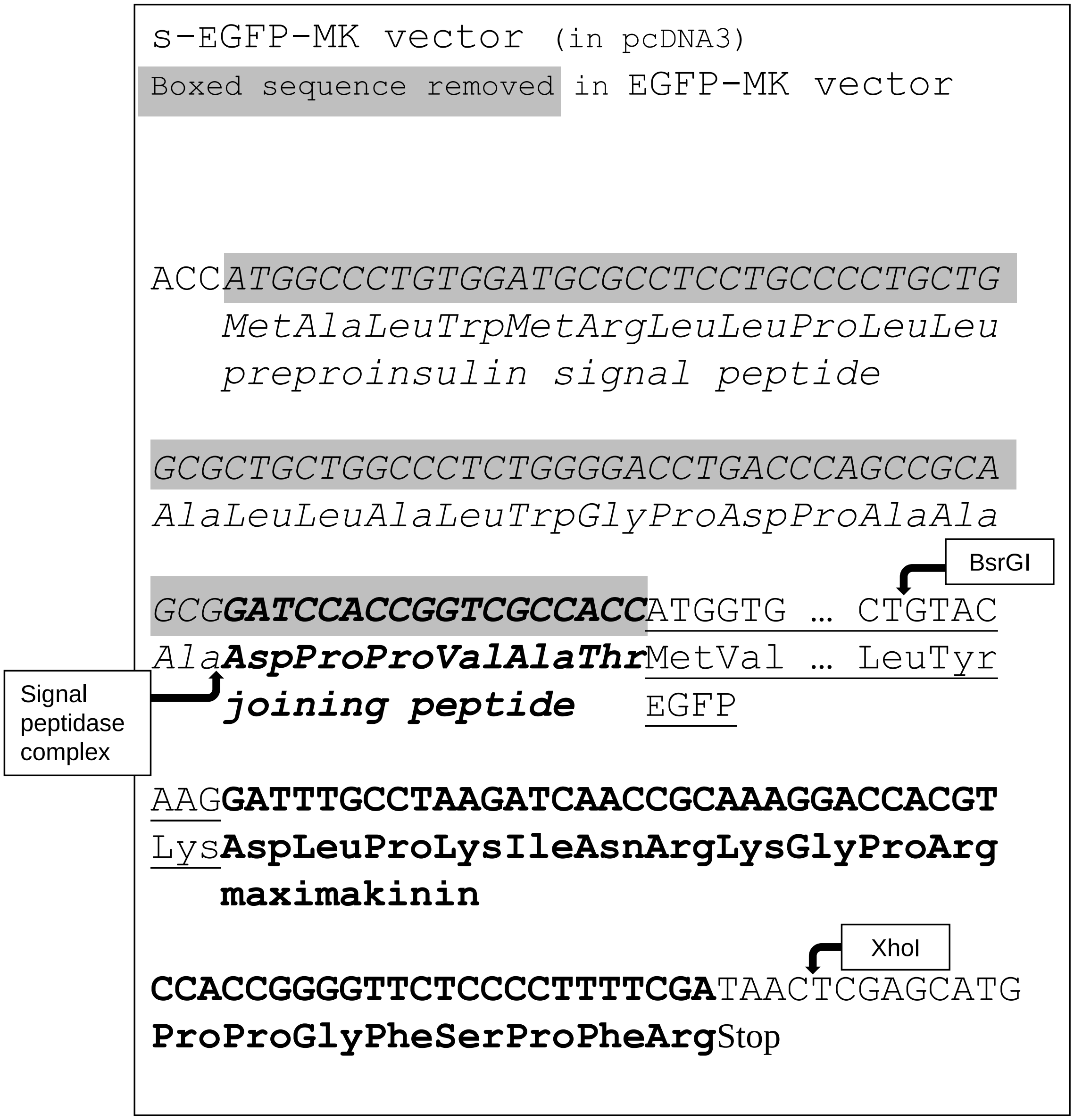

Supplementary Figure 2. Partial coding sequence of the s-EGFP-MK and the EGFP-MK vectors, the second being obtained following the deletion of the boxed sequence (signal peptide + joining peptide). See text for description.

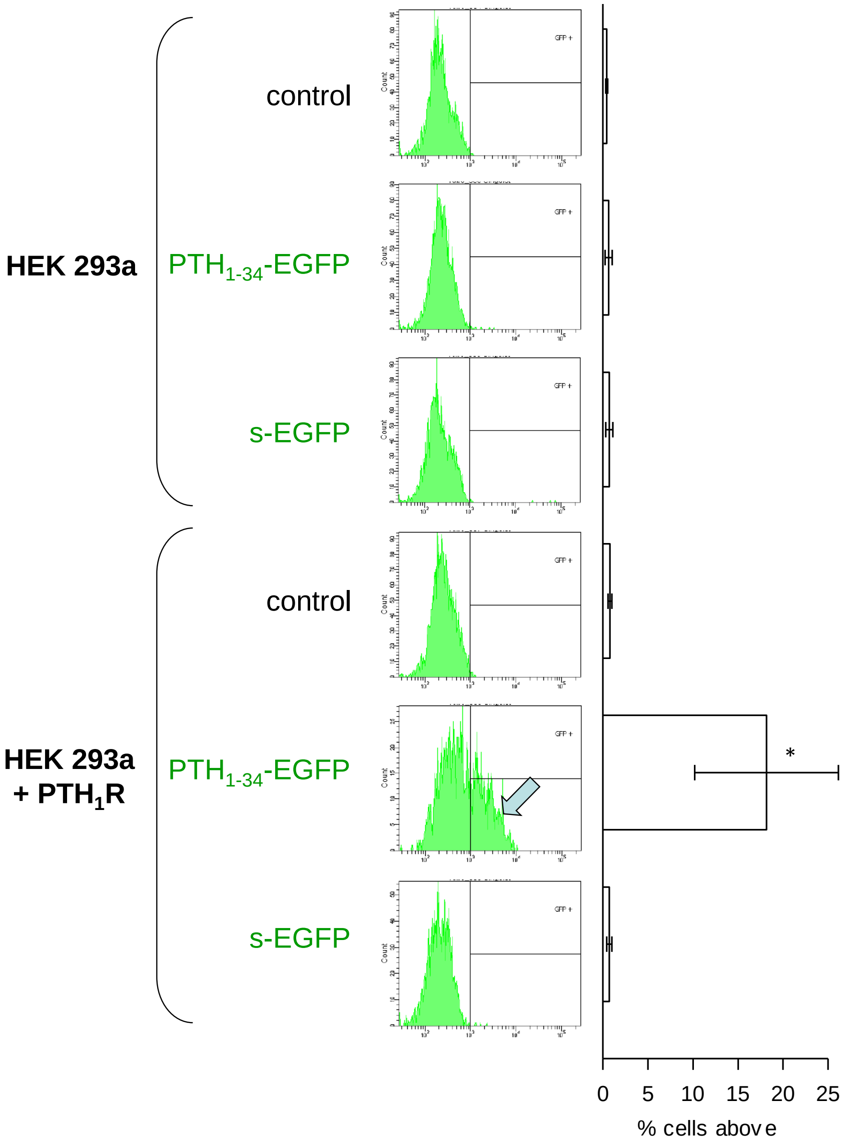

Supplementary Figure 3. Cytofluorometry of HEK 293a cells that optionally expressed PTH_1R (3 conditions at bottom), sequentially detached and stained for 30 min (37ºC) with the undiluted conditioned medium of other cells producing s-EGFP or $PTH_{1\text{-}34}$-EGFP, as indicated. Left: distributions based on the counting of 10000 cells. A threshold of autofluorescence was defined using control cells with no fluorophore. It was surpassed only under one set of experimental conditions (arrow). Right: proportion of cells above the threshold under each experimental condition in 3 separate experiments (means ± s.e.m.). ANOVA indicated that the values were heterogeneous ($P<0.02$). * $P<0.05$ vs. controls with or without receptors (Tukey-Kramer multiple comparison test). s-EGFP did not significantly stain cells.

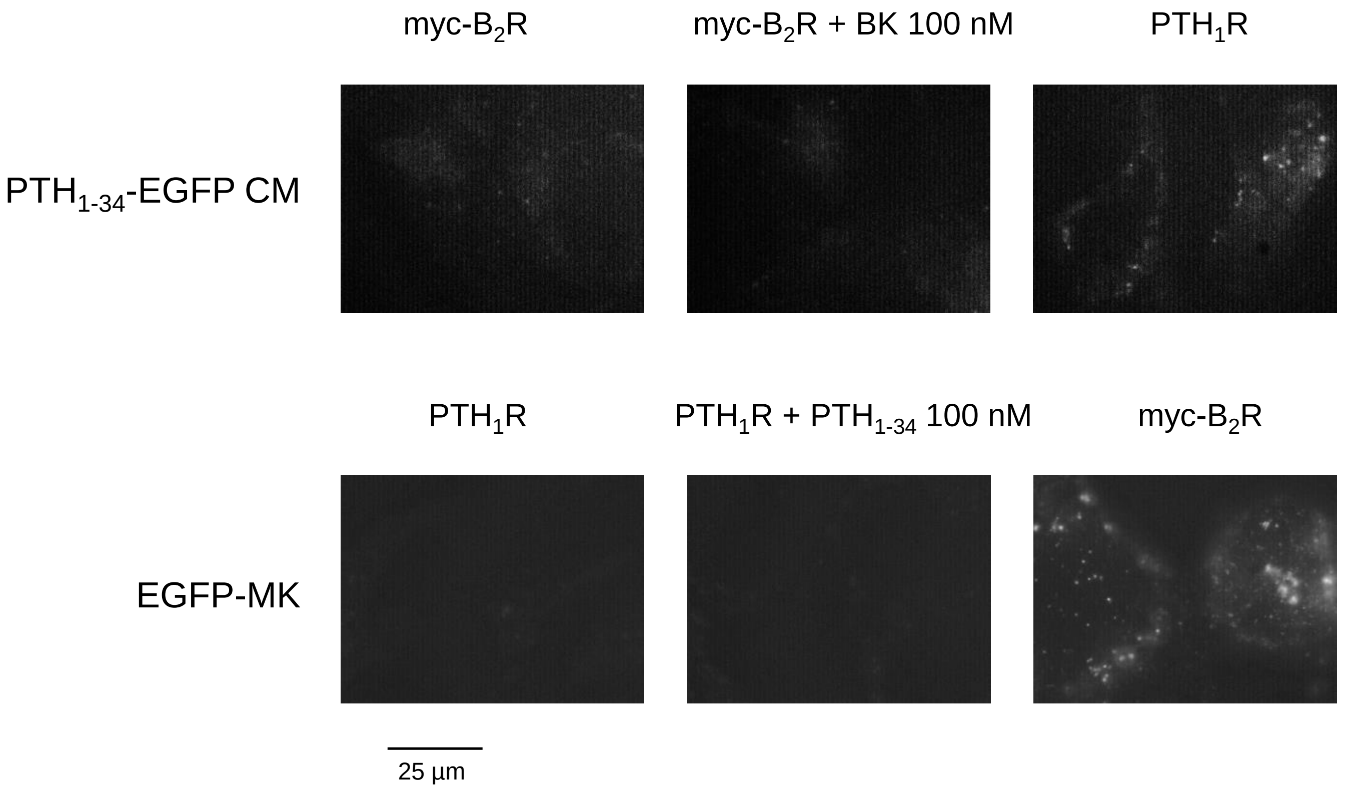

Supplementary Figure 4. Lack of stimulated uptake of fluorescent probes by cells expressing the non-corresponding receptor. HEK 293a cells expressing the indicated receptor were incubated for 30 min with one of the EGFP fusion protein and optionally stimulated with a non-fluorescent peptide, as indicated.
